# Inhibition of GPR30 by estriol prevents growth stimulation of triple-negative breast cancer cells by 17β-estradiol

**DOI:** 10.1186/1471-2407-14-935

**Published:** 2014-12-11

**Authors:** Rainer Girgert, Günter Emons, Carsten Gründker

**Affiliations:** Department of Obstetrics and Gynecology, University Medicine Göttingen, Robert-Koch-Strasse 40, D-37075 Göttingen, Germany

**Keywords:** Triple-negative breast cancer, Targeted therapy, GPR30, Estriol, Signal transduction

## Abstract

**Background:**

Due to the lack of ERα, triple negative breast cancers (TNBCs) are not susceptible to endocrine therapy using antiestrogens. However, the majority of TNBCs express the membrane bound estrogen receptor GPR30. We have recently shown that knock-down of GPR30 expression prevented growth stimulation of TNBC cell lines by 17β-estradiol. Now we analyzed whether specific inhibition of GPR30 represents a new option for therapy of TNBC.

**Methods:**

Growth of TNBC cells was assessed using Alamar-blue colorimetric assay. Activation of c-Src and EGF-receptor was assessed using Western blots. Expression of c-fos, cyclin D1 and aromatase was quantified by RT-PCR. Gα-specific signaling of GPR30 was analyzed by electrophoretic mobility shift assay.

**Results:**

HCC1806 cells showed the highest GPR30 expression, in HCC70 cells it was clearly lower, in MDA-MB-231 cells it was lowest. 10^-8^ M 17β-estradiol significantly increased proliferation of HCC1806 cells to 134 ± 12% of control (p < 0.01). Proliferation of HCC70 cells was slightly increased to 116 ± 8% of control. Estriol significantly reduced cell number of HCC1806 cells to 16 ± 12% (p < 0.01). Cell number of HCC70 cells and of MDA-MB-231 cells was reduced to 68 ± 25% and to 61 ± 10%, respectively.

Activity of Src kinase increased to 150 ± 10% (p < 0.05) by 10^-8^ M 17β-estradiol treatment in HCC1806 and to 220 ± 20% in HCC70 cells (p < 0.01). Estriol treatment completely inhibited 17β-estradiol-induced p-src activation. Transactivation of EGF-receptor increased by estradiol treatment to 350% in HCC1806 and to 280% in HCC70 cells. Estriol completely suppressed EGF-receptor transactivation. *c-fos* expression increased to 260% and to 190%, respectively. Estriol reduced this induction to 160% (HCC1806) and below control in HCC70 cells. Cyclin D1 was induced to 290% (HCC1806) and 170% (HCC70) and completely inhibited by estriol. 17β-estradiol increased CREB-phosphorylation to 400%. Binding of phospho-CREB to a CRE of cyclin D1 was enhanced to 320%.

**Conclusion:**

Specific pharmacological inhibition of GPR30 might become a promising targeted therapy for TNBC in future.

**Electronic supplementary material:**

The online version of this article (doi:10.1186/1471-2407-14-935) contains supplementary material, which is available to authorized users.

## Background

Breast cancer is the most frequent cause of mortality from cancer in women. Therapy of ERα-positive tumors using anti-estrogens, like Tamoxifen and aromatase inhibitors achieves an overall survival of about 82% after eight years [1]. Triple-negative breast cancers (TNBCs) that do not express ERα and progesterone receptors and do not overexpress Her-2neu gene product are not susceptible to endocrine therapy. Mortality of patients with TNBC is double as high as for carriers of ERα-positive tumors [1]. For this reason, there is an urgent need for development of innovative, targeted therapies for this group of patients.

In the last years a number of new therapeutic approaches were tested with limited success. Treatment with platinum compounds resulting in a response rate of 30% could be increased to 49% by the combination with Cetuximab, an antibody to the epidermal growth factor receptor [2]. The DNA-repair enzyme, poly-ADP-ribose polymerase (PARP), was also found to be a promising target in TNBC [3, 4].

For many years, it was assumed that an estrogen receptor resides at the plasma membrane. G-protein coupled receptor 30 (GPR30) was identified to be responsible for most rapid signaling events of 17β-estradiol [5, 6]. Before identification of GPR30 as third kind of estrogen receptors, other authors supposed that rapid estrogen signaling is initiated by a divergent membrane bound ERα [7]. GPR30 expression is prevalent in TNBC and associated with a higher recurrence rate [8].

In early experiments, almost before function of GPR30 was described, a rapid increase of cAMP was observed after stimulation of MCF-7 breast cancer cells with 17β-estradiol [9]. Only some years later it was discovered, that binding of 17β-estradiol to GPR30 increases adenylate cyclase activity and MAP-kinase Erk 1 [6, 9, 10]. Increased cAMP leads to phosphorylation of CREB that subsequently binds to cAMP-response elements (CRE) on promoters of mitogenic genes [11]. Activation of MAP-kinase finally leads to enhanced proliferation of breast cancer cells. The signaling via βγ-subunits in TNBC has been briefly described [12]. In addition to 17β-estradiol, selective estrogen receptor modulator Tamoxifen and complete ERα antagonist Fulvestrant bind to GPR30 and activate certain signaling pathways in breast cancer cells, thus leading to stimulation of proliferation [5]. According to these observations GPR30 has been proposed to be an excellent new therapeutic target for the treatment of TNBC [13].

Recently, we reported that in TNBC cell lines an increase of proliferation by17β-estradiol was dependent on GPR30, as it was completely prevented by knock-down of GPR30 expression using specific siRNA [12].

Dennis et al. developed G15, a specific inhibitor of GPR30 signaling [14]. In addition, estriol has been shown to be a potent inhibitor of GPR30 [15]. In this report we have analyzed the efficacy of estriol as inhibitors of GPR30 on growth inhibition of TNBC cells. Similar attempts using G15 in TNBC were less successful than with estriol and are additionally presented as supplementary material.

## Methods

### Cell lines, chemicals and cell culture

TNBC cell lines HCC1806, HCC70 and MDA-MB-231 were obtained from American Type Culture Collection (ATCC, Manassas, Virginia, USA). In order to guarantee the identity of the cell lines over the years, cells were expanded after purchase and aliquots were stored in liquid nitrogen. Every half year a new frozen stock was opened and expanded to carry out the experiments. Cells were cultured in MEM (Biochrom, Berlin, Germany) supplemented with 2 mM glutamine, 6 ng/ml insulin, 10 ng/ml transferrin, penicillin (50 U/ml), streptomycin (50 μg/ml) from Gibco (Paisley, UK), and 5% fetal bovine serum (Biochrom, Berlin).

17β-estradiol (E2), estriol, insulin, and transferrin were from Sigma-Aldrich (Deisendorf, Germany). G15 was purchased from R & D systems (Wiesbaden, Germany). Primers for PCR and biotin-labeled oligonucleotide probes for electrophoretic mobility shift assay were produced by MWG-eurofins (Ebersberg, Germany).

### Proliferation assays

Proliferation assays for 17β-estradiol in the absence and in the presence of estriol were performed in phenol red-free medium supplemented with charcoal depleted serum (CD-FCS) as previously described [16]. CD-FCS was prepared according to the procedure described by Stanley et al. [17].

Proliferation assays were performed at least three times in quadruplicates with different passages. Means and standard deviations of the optical density (OD) of the replicates were calculated.

### Treatment of cells

For stimulation of TNBC cells to analyze signal transduction of GPR30, 4×10^6^ cells were plated in culture medium into 25 cm^2^-culture flasks. After attachment, cells were serum starved for 24 hours to synchronize the 17β-estradiol-starved cells in G_0_-phase. Serum starved cells were treated for 30 minutes either with 10^-4^ M estriol or solvent (0.1% ethanol) and subsequently stimulated with 10^-8^M 17β-estradiol in 0.1% ethanol for 10 min or 20 minutes. Cells were harvested and cell pellets lysed in 100 μl Cell lytic M (Sigma, Deisendorf, Germany), supplemented with protease-inhibitor (Sigma, Deisendorf, Germany) and phosphatase-inhibitor (Sigma, Deisendorf, Germany).

### Western blots

Lysates of cells were cleared at 15000 g for 5 minutes and the protein concentration in the supernatant was determined using the method of Bradford. 50 μg of each sample were separated in a 7.5% polyacrylamid gel, blotted on PVDF-membrane and sequentially detected with rabbit-anti-human primary antibodies. GPR30 expression was detected with anti-GPR30 (sc-48524) from Santa Cruz (Dallas, TX), anti-phospho-Src (2113), anti-Src (2109), anti-S133phospho-CREB (9198), were all purchased from Cell Signaling and anti-CREB (04-767) from Millipore. Antibody to phospho Tyr^1173^EGF-receptor (324864) was from Calbiochem (Darmstadt, Germany), anti-EGF-receptor antibody (2235) from Epitomics (Hamburg, Germany) and anti-actin from Sigma Chemicals (Deisendorf, Germany). All primary antibodies were used diluted 1:2000 in TBST.

After washing in TBST blots were incubated with a 1:20.000 dilution of horseradish peroxidase conjugated goat-anti-rabbit antibody (ECL, GE-Healthcare, Freiburg, Germany). After washing, blots were incubated with the chemoluminescence reagent Femto (Thermo-Scientific, Rockford, IL) and scanned on a Licor C-digit chemoluminescence detector (Licor, Lincoln, NE). Densitometric evaluations of the protein bands were performed using the analysis tool of the Image Studio Digits Vers.4 delivered with the Licor C-Digit chemoluminescence detector (Licor, Lincoln, NE) and were normalized to actin.

### Analysis of GPR30 signaling

GPR30 signal transduction and gene expression of c-fos, cyclin D1 and aromatase were analyzed as previously described [12].

In detail, from TNBC cells, pretreated with estriol or not and subsequently stimulated for 30 minutes with 10^-8^M 17β-estradiol, RNA was purified using the RNeasy-kit (Qiagen, Hilden, Germany).

200 ng of each RNA were transcribed using 400 u Superscript reverse transcriptase (Invitrogen, Karlsruhe, Germany) in the presence of 0.5 μM oligo-dT primer for 60 min at 37°C. 5 μl of the resulting cDNA were amplified with 1 u Taq polymerase (Peqlab, Erlangen, Germany) in the presence of 200 μM dNTPs and 200 nM of the appropriate primers described in [12].

Optimal PCR conditions for each gene were ascertained, guaranteeing that generation of the PCR-products was in the exponential phase. Therefore cDNA of c-fos was amplified by 32 cycles, cyclin D1 by 35 cycles, and aromatase by 28 cycles. As reference the RNA of the ribosomal protein L7 was amplified by 20 cycles.

PCR-products were separated in a 2% agarose gel (Type IV, special high EEO, Sigma Chemicals, Steinheim, Germany) in 0.5x TBE buffer at 100 V for 30 min. Gels were stained in ethidium bromide (2 μg/ml) for 30 min and photographed on a transiluminator using a CDS camera (TD20, Kodak, Rochester).

### Electrophoretic mobility shift assay

The effects of estriol on the downstream signaling of GPR30 were analyzed using an electrophoretic mobility shift assay (EMSA) as previously described [18] using oligonucleotides representing the promoter sequence from -14 to +11containing a CRE (bold) labeled at the 3’end by Biotin.

Cyclin D1-sense: AACAACAGTA**ACGTC**ACACGGACTA.

Cyclin D1-antisense: TTGTTGTCAT**TGCAG**TGTGCCTGA.

### Statistical analysis

The data were tested for significant differences by one-way analysis of variance followed by Student–Newman–Keuls’ test for comparison of individual groups, after a Bartlett test had shown that variances were homogenous.

## Results

### Comparison of GPR30 expression in triple-negative breast cancer cell lines

**Figure 1:**
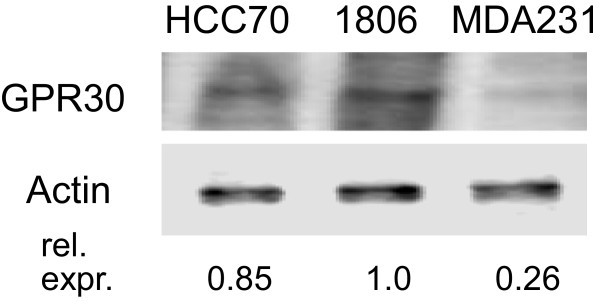
**GPR30 expression in TNBC cell lines used.** 10 μg protein extracts of HCC70-, HCC1806- and MDA-MB-231-cells were separated in a polyacrylamide gel and Western-blots were sequentially analyzed using antibodies against GPR30 and actin (house-keeping gene). GPR30 expression was determined by densitometry of the bands giving the relative expression in each cell line.

Protein extracts of three different TNBC cell lines were analyzed on Western-blots for expression of GPR30 (Figure 1). We proved specifity of the used antibody by knocking down GPR30 with siRNA in HCC1806 and HCC70 [12]. GPR30 was most prominent in HCC1806 cells. HCC70 cells expressed about 85% of the amount of GPR30 detected in HCC1806. Using immunohistochemistry TNBC cell line MDA-MB-231 presented as GPR30 negative [19], but our Western-blot analysis revealed traces of GPR30 protein in this cell line accounting for 26% of the amount present in HCC1806 (Figure 1).

### Stimulation of cell proliferation by 17β-estradiol in TNBC cells is prevented by estriol

**Figure 2:**
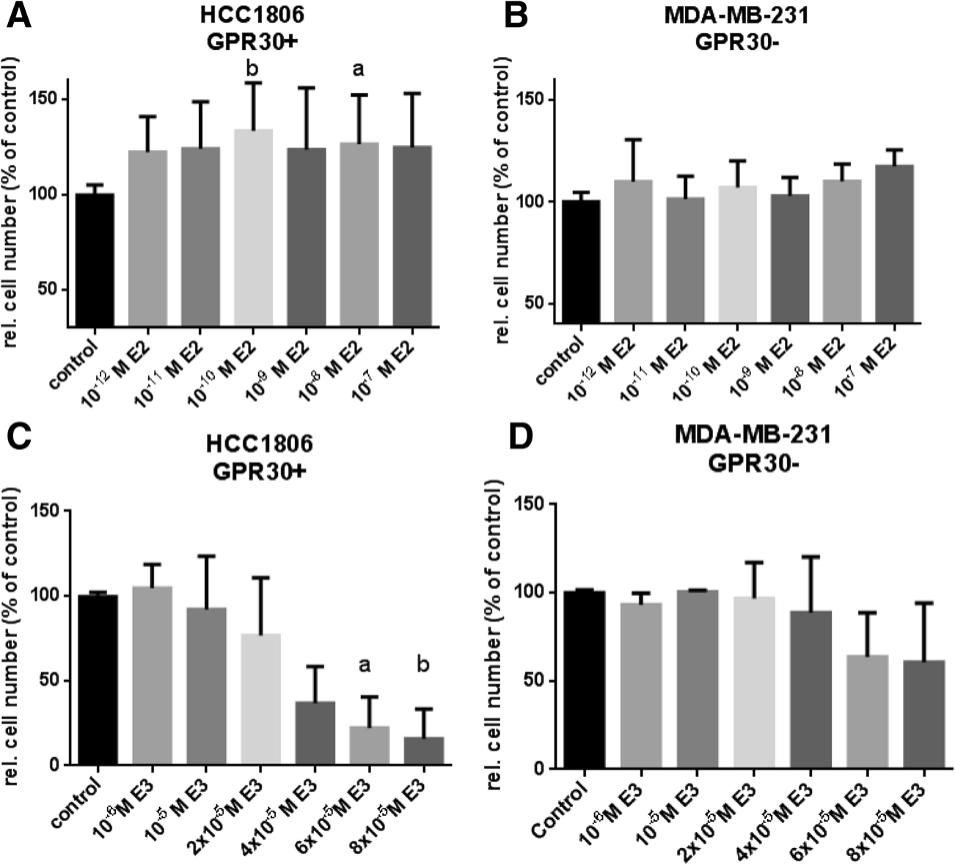
**Impact of 17β-estradiol and estriol on proliferation of TNBC cells.** Cells of a GPR30 positive (HCC1806) and a GPR30 negative TNBC cell line (MDA-MB-231) were grown for 7 days in culture medium supplemented with charcoal-treated FCS either in the presence of 10^-12^ M to 10^-6^ M 17β-estradiol **(A and** **B)** or 10^-6^ M to 8×10^-5^ M estriol **(C and** **D)**. Cell number was evaluated in microwell plates by a colorimetric assay using Alamar blue. Optical densities (OD) measured in the non-stimulated wells (control) were set 100%. The ODs estimated in the stimulated wells were divided by the average value of the control wells to give the relative cell number in % of control achieved under the indicated conditions. (a) p < 0.05 vs. control; (b) p < 0.01 vs. control. Data are mean values and SE of three independent experiments with four replicates.

The effects of 17β-estradiol and estriol on proliferation of TNBC cell lines with high-level GPR30 expression (HCC1806) and low-level GPR30 expression (MDA-MB-231) were analyzed over a concentration range between of 10^-12^ M and 10^-6^ M 17β-estradiol (Figure 2A and B) and with estriol between 10^-6^ M and 8x10^-5^ M (Figure 2C and D). Cell number of the TNBC cell line HCC1806 slightly increased in the presence of increasing concentrations of 17β-estradiol reaching a plateau of 130% between 10^-10^ M and 10^-8^ M estradiol. Even in charcoal stripped medium without estradiol HCC1806 cells proliferated, due to growth factors present in the charcoal stripped serum (Additional file 1). In MDA-MB-231 cells, reported to be negative for GPR30, there was no significant elevation of cell number observed at any of the tested estradiol concentrations (Figure 2B). The traces of GPR30 detected on Western-blot of MDA-MB-231 (Figure 1) were not sufficient for 17β-estradiol to increase cellular growth of this TNBC cell line. With increasing concentrations estriol reduced cell number of 17β-estradiol stimulated HCC1806 cells very clearly down to 16 ± 12% (p < 0.01) at 8× 10^-5^ M whereas in MDA-MB-231 cells expressing only low amounts of GPR30 cell number was reduced to only 61 ± 10% at the highest applied concentration. In HCC70 cells expressing less GPR30 than HCC1806 (Figure 1) the reduction of cell number was lower to 68 ± 25% (Data not shown).

**Figure 3:**
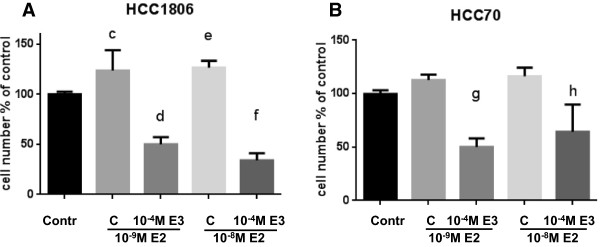
**Comparison of the inhibitory effect of estriol on the proliferative effect of 17β-estradiol on TBNC cells.** Cells of two GPR30 expressing TNBC cell lines **(A)** HCC1806, **(B)** HCC70 were stimulated with 10^-9^ M E2 or 10^-8^ M E2 in the absence or presence of 10^-4^ M E3. Cell number was evaluated using Alamar blue. ODs estimated in stimulated wells were divided by the average value of the control wells to give the relative cell number in % of control achieved under the indicated conditions. (c) p < 0.01 vs. control; (d) p < 0.001 vs. 10^-9^ M E2 w/o E3; (e) p < 0.01 vs. control; (f) p < 0.001 vs.10^-8^ M E2 w/o E3; (g) p < 0.01 vs. 10^-9^ M E2 w/o E3; (h) p < 0.05 vs. 10^-8^ M E2 w/o E3. Data are mean values and SE of three independent experiments with four replicates.

To evaluate the inhibitory activity of estriol in the presence of 17β-estradiol growth of TNBC cells was treated either with 17β-estradiol alone or in combination with 10^-4^ M estriol (Figure 3A and B). Cell number of HCC1806 cells increased at 10^-8^ M 17β-estradiol to 127 ± 8% of control. In cells pretreated with 10^-4^ M estriol cell number significantly decreased to 54 ± 7% of control (p < 0.05) despite stimulation with 10^-8^M 17β-estradiol (Figure 3A). In cell line HCC70 10^-8^ M 17β-estradiol increased cell number to 116% of control, co-treatment with 10^-4^ M estriol significantly decreased cell number to 64% of control (p < 0.01) (Figure 3B). Estriol clearly prevented the stimulation of proliferation by 17β-estradiol in both TNBC cell lines. Antiproliferative effects of estriol paralleled the amount of GPR30 expressed in the various cell lines (Figure 1).

### Estriol inhibits GPR30 dependent transactivation of EGF-receptor

**Figure 4:**
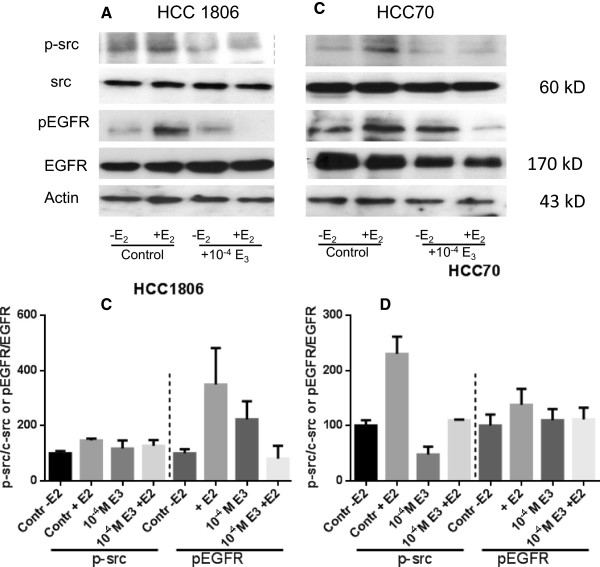
**Disruption of signal transduction of GPR30 in TNBC cell lines.** Western blots **(A)** HCC1806 and **(B)** HCC70. Serum starved cells (lane 1) were stimulated for five minutes with 10^-8^ M 17β-estradiol (lane 2) or pretreated for 30 minutes with 10^-4^ M estriol E3 (lane 3) and E3 treated cells were subsequently stimulated with 10^-8^ M 17β-estradiol (lane 4). Cells were lysed, proteins separated in a polyacrylamide gel, blotted onto a PVDF-membrane, and the indicated proteins were sequentially detected with antibodies against phospho-src (panel 1), total-src (panel 2), phospho-EGF-receptor (panel 3), total EGF-receptor (panel 4), and actin, as housekeeping gene (panel 5). Representative results of three independent preparations. **(C)** and **(D)** densitometric evaluation of Western blot results of p-src and pEGFR in HCC1806 cells **(C)** and HCC70 **(D)**.

In order to explain the effects of estriol on proliferation of TNBC cells activation of c-src and EGF-receptor by 10^-8^ M 17β-estradiol in the absence and in the presence of 10^-4^ M estriol was analyzed. In serum-starved HCC1806 cells phosphorylation of c-src at Tyr^416^ was detectable. Activation of c-src was maximal after 5 minutes of stimulation with 10^-8^ M 17β-estradiol (Figure 4) and decreased after 15 minutes of stimulation (not shown). Activation of p-src increased to 150 ± 10% (p < 0.05) after 5 minutes stimulation with 17β-estradiol (Figure 4A, lane 2). In HCC70 cells p-src increased to estimated 220 ± 20% of control (p < 0.01) (Figure 4B, lane 2). In HCC1806 cells pretreated for 30 minutes with 10^-4^ M estriol activation of src-kinase by 17β-estradiol was completely blocked (Figure 4A, lane 4); in HCC70 cells after pretreatment with estriol no p-src activation by 10^-8^ M 17β-estradiol could be observed (Figure 4B, lane 4).

Activation of EGF-receptor downstream of p-src was increased to 350 ± 160% (p < 0.01) in HCC1806 cells by 17β-estradiol (HCC70: 280 ± 80%, p < 0.01). Pretreatment of the cells with 10^-4^ M estriol completely prevented activation of EGF-receptor by 17β-estradiol in both cell lines (Figure 4A and B, lane 4).

### Estriol prevents induction of c-fos expression by 17β-estradiol

**Figure 5:**
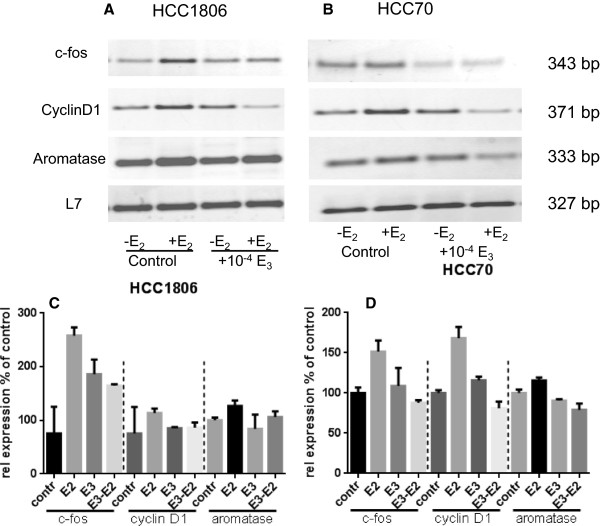
**Prevention of gene expression by inhibition of GPR30 with estriol.** RT-PCR of **(A)** HCC1806 and **(B)** HCC70 Serum starved cells of TNBC cell lines (lane 1) were stimulated for 30 minutes with 10^-8^ M 17β-estradiol (lane 2) or pretreated with 10^-4^ M estriol (lane 3) and subsequently stimulated with 10^-8^ M 17β-estradiol (lane 4). mRNA was extracted, transcribed to cDNA and amplified by PCR using primers specific for c-fos (panel 1), cyclin D1 (panel 2) or aromatase (panel 3). L7, a ribosomal housekeeping gene, was amplified to prove the presence of equal amounts of RNA in each PCR reaction of the respective cell line (panel 4). Representative results of three separate experiments. **(C)** and **(D)** densitometric evaluation of RT-PCR results of c-fos, cyclin D1 and aromatase in HCC1806 cells **(C)** and HCC70 **(D)**.

Following activation of EGF-receptor the growth promoting signal is forwarded via MAP-kinase Erk to the nucleus where finally expression of c-fos is induced. Serum-starved cells of two different TNBC cell lines (HCC1806 and HCC70) were stimulated for 30 minutes with 10^-8^ M 17β-estradiol. mRNA of these cells was analyzed for c-fos expression by RT-PCR and expression of c-fos was compared to the expression in non-stimulated cells (Figure 5). Stimulation by 10^-8^ M 17β-estradiol most strongly increased c-fos expression in HCC1806 cells to 260 ± 20% (p < 0.001) (Figure 5A, lane 2). In HCC70 cells stimulated with17β-estradiol c-fos expression accounted for 190 ± 70% of control (Figure 5B, lane 2). If the HCC 1806 cells were pretreated with 10^-4^ M estriol for two hours 17β-estradiol increased c-fos expression only to160 ± 3% (p < 0.01). In HCC70 cells pretreated with estriol the increase of c-fos expression by 17β-estradiol was attenuated below control value.

### Induction of cyclin D1 expression by 17β-estradiol is inhibited by estriol

Cyclin D1 is necessary for transfer of cells from G1-phase to S-phase of cell cycle and a prerequisite for proliferation. Therefore, cyclin D1 expression was analyzed in TNBC cell lines after stimulation of the cells with 10^-8^ M 17β-estradiol for 30 minutes (Figure 5A and B, lane 2). In HCC1806 cells 17β-estradiol increased cyclin D1 expression to 300 ± 30% of control (p < 0.05) and estriol completely prevented activation of cyclin D1 expression (Figure 5A, lane 4). Stimulation of HCC70 cells with 17β-estradiol lead to a 170 ± 20% expression of cyclin D1 (p < 0.01) (Figure 5B, lane 2), that was completely abolished by pretreatment with 10^-4^ M estriol (Figure 5B, lane 4).

### Aromatase expression in TNBC cells

Aromatase, an enzyme necessary for synthesis of 17β-estradiol, was reported to be GPR30 dependently regulated [20]. RT-PCR analysis of the mRNA of TNBC cell lines revealed an extraordinary high expression of aromatase in HCC1806 cells (Figure 5A). This high expression of aromatase was further increased to 130% after treatment with 10^-8^ M 17β-estradiol. In HCC70 cells expressing much less aromatase (Figure 5B, lane 1) estradiol significantly stimulated expression of aromatase to 120 ± 5% (p < 0.05) (Figure 5B, lane 2). A further TNBC cell line tested (MDA-MB-435) showed a more pronounced induction of aromatase to 180 ± 20% (p < 0.01) of control after stimulation with 10^-8^ M 17β-estradiol (data not shown). In all three cell lines pretreatment with 10^-4^ M estriol blocked estradiol-induced stimulation of aromatase expression (Figure 5A and B, lane 4).

### Gα-dependent signaling of GPR30

#### Phosphorylation of CREB-protein

**Figure 6:**
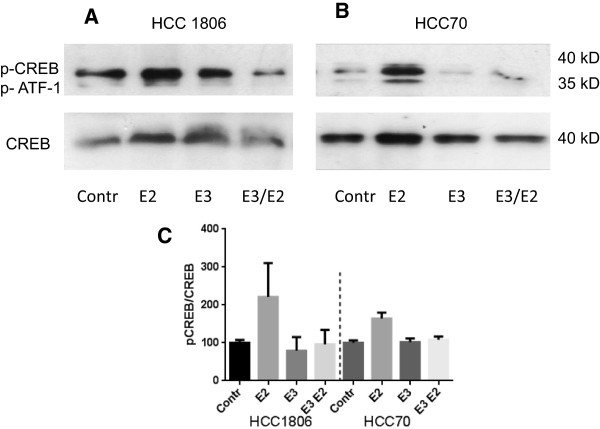
**Phosphorylation of CREB protein in TNBC cells.** HCC1806 cells **(A)** and HCC70 cells **(B)** serum starved for 24 h (lane 1) were stimulated for 30 minutes with 10^-8^ M 17β-estradiol (lane 2) or pretreated for 30 minutes with 10^-4^ M estriol E3 (lane 3) and E3 treated cells were subsequently stimulated for 30 minutes with 10^-8^ M 17β-estradiol (lane 4). Cells were lysed, proteins separated in a polyacrylamide gel, blotted onto a PVDF-membrane and phospho-CREB and total CREB proteins were sequentially detected with according antibodies. Representative results of three independent preparations. **(C)** densitometric evaluation of Western blots results of CREB phosphorylation under various conditions normalized to total CREB expression in HCC1806 cells and HCC70.

Activation of GPR30 by its ligands, 17β-estradiol, 4-Hydroxytamoxifen or Fulvestrant releases of Gα from the heterotrimeric G-protein complex after ligand binding and further initiates activation of adenylyl cyclase PKA and phosphorylation of the cAMP-responsive element binding protein CREB [21]. Phosphorylation of CREB after stimulation of TNBC cells with 17β-estradiol was analyzed using Western blot technology (Figure 6).

Two protein bands at 35 kD and 40 kD became visible in the variously treated samples using a phospho-Ser^133^-CREB antibody (Cell Signaling, Boston, MA). According to the manufacturer this antibody cross-reacts with phospho-ATF-1, a CREB-related protein, that gives rise to the lower weight band [22]. Even in serum starved cells of HCC1806 a substantial CREB phosphorylation was detectable (Figure 6A, lane 1). Stimulation of HCC1806 cells with 10^-8^ M 17β-estradiol for 30 minutes led to a slightly increased phosphorylation of CREB (Figure 6A, lane 2) Stimulation of HCC1806 cells resulted in an increase of phosphorylated CREB to 220 ± 110% of control (Figure 6B, lane 2). Pretreatment of the cells with 10^-4^ M estriol for 2 hours lead to a slight decrease of phospho-CREB (Figure 6B, lane 3). If HCC70 cells pretreated with estriol were subsequently stimulated with 10^-8^ M 17β-estradiol phosphorylation of CREB was clearly reduced even below control level (Figure 6B, lane 4).

### Estriol reduces binding of CREB protein to the promoter of cyclin D1

**Figure 7:**
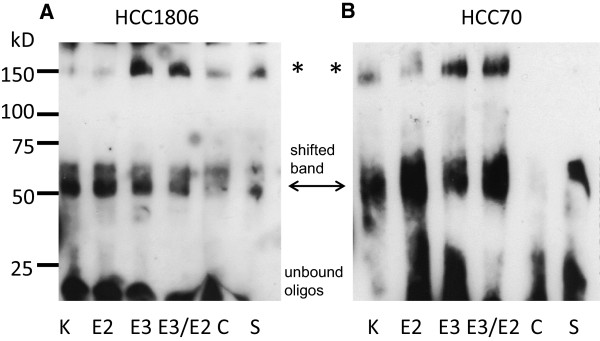
**Electrophoretic mobility shift of a cAMP responsive element from cyclin D1 promoter.** Cells of TNBC cell line HCC1806 **(A)** or of cell line HCC70 **(B)** were serum starved (lane 1) and either stimulated for 15 minutes with 10^-8^M 17β-estradiol (lane 2), or pretreated for 30 minutes with 10^-4^ M estriol (lane 3) before they were stimulated with 10^-8^M 17β-estradiol for further 15 minutes (lane 4). Cells were harvested and nuclear proteins purified from the cell pellets were incubated with the cyclin D1 specific oligonucleotide probe for 20 minutes at room temperature. After binding reaction the mixture was separated on a 6% non-denaturing polyacrylamide gel, blotted onto a nylon membrane and the biotin-labeled probe was detected using HRP-labeled streptavidin. To prove specificity of reaction a control containing a 100-fold excess of unlabeled oligonucleotide was added to a second estradiol-treated sample (lane 5) and for supershift (S) 1 μl phospho-CREB antibody was added (lane 6). Representative EMSA-assay of three independent experiments.

Phosphorylated CREB binds to the cAMP responsive elements in promoters of genes and initiates their transcription. To study the influence of 17β-estradiol on binding of phosphorylated CREB to CRE of the cyclin D1 promoter nuclear proteins were purified from estradiol stimulated HCC1806 and HCC70 cells and used for an electrophoretic mobility shift assay (Figure 7).

The non-bound oligonucleotides were moving at the front of the gel in all samples. There is a clearly visible band of the biotin-labeled oligonucleotide probe containing a CRE from the cyclin D1 promoter at about 50 kD in all lanes except lane 5, where a 100-fold excess of unlabeled probe was additionally added to the nuclear proteins. Even in serum starved cells a weak shifted band was detected (Figure 7, lane 1). In HCC1806 cells stimulated with 10^-8^ M 17β-estradiol the intensity of the shifted band increased on average to 161 ± 59% (Figure 7A, lane 2) and 320 ± 170% of control in HCC70 cells (Figure 7B, lane 2), whereas in HCC70 cells pretreated with estriol the CREB-signal was only 30% stronger than in serum starved cells after stimulation with 17β-estradiol (Figure 7B, lane 4). Besides the band at 40 kD specific for CREB an additional shifted band occur on the blotted gel at about 150 kD that is either shifted by other unknown nuclear proteins or shifted by CREB additionally complexed with further yet unidentified proteins.

An electrophoretic mobility shift assay was also performed using an oligonucleotide representing a DNA-sequence of a CRE detected further upstream of the above mentioned CRE at position -517 to -493. In none of the TNBC cell lines we observed a shifted band of this upstream CRE after stimulation of the cells with 10^-8^ M 17β-estradiol (data not shown).

## Discussion

TNBC is characterized by the lack of expression of estrogen receptor α (ERα) and progesterone receptors and no overexpression of Her-2. This makes these tumors refractory for standard antiestrogen therapy with Tamoxifen and for antibody therapy using Trastuzumab. GPR30, a membrane-bound receptor for estrogens responsible for fast non-genomic effects of 17β-estradiol, might be a promising target in TNBC. Immunohistochemical staining of sections of a small cohort of TNBCs for GPR30 revealed that most of these tumors strongly expressed GPR30. GPR30 expression correlated with a higher recurrence rate of TNBC [8]. Experimentally, it has been shown that 17β-estradiol also induces proliferation in immortalized breast epithelial cell line MCF10A in a GPR30-dependent manner as well as does the GPR30-specific agonist G1 [23]. Only recently, we have used GPR30 specific siRNA to knock-down GPR30 expression in TNBC making the treated cells refractory to growth stimulation by 17β-estradiol [12]. There are meanwhile a number of clinical investigations discussing an involvement of GPR30 in malignant transformation of breast cancer cells [24–26]. In addition, GPR30 was found to play a growth promotion role in ovarian cancer [27]. All these observations strongly support our assumption that stimulation of GPR30 by circulating 17β-estradiol contributes to the malignant behavior of TNBC. GPR30 was also expressed in the TNBC cell lines used in the present study, except MDA-MB-231 that were used as negative control (Figure 2B and D). Stimulation of TNBC cell lines with the ligands of GPR30, 17β-estradiol and 4-Hydroxy-tamoxifen leads to activation of src-kinase and EGF-receptor phosphorylation. Recently we reported that knock-down of GPR30 expression using siRNA completely prevented activation of these kinases and abrogated the stimulation of cell proliferation by 17β-estradiol in TNBC cell lines [12]. Therefore, a search for potent pharmacological inhibitor of GPR30 might prove valuable for the treatment of TNBC.

G15, a substance from a compound library, was reported to be selective for GPR30 and inhibited signal transduction of GPR30 [14]. The naturally occurring estrogen metabolite estriol has also been shown to be a GPR30 antagonist in estrogen receptor-negative breast cancer cell line SKBr3 [15]. SKBr3 is not a representative of TNBC cell lines, because this cell line overexpresses Her-2. But as we show in the present report, estriol is a potent GPR30 antagonist in a number of TNBC cell lines. Treatment of TNBC cell lines with estriol led to a significant reduction in cell number to 16% of control at 8×10^-5^ M estriol, the highest concentration achievable in aqueous solution. The inhibitory effect of estriol on cell proliferation correlated with the amount of GPR30 expressed in the various TNBC cell lines.

Phosphorylation of src-kinase and transactivation of EGF-receptor by 17β-estradiol were almost as successfully prevented as was observed after knock-down of GPR30 using siRNA [12]. In addition to the inhibition of EGF-receptor activation, estriol also proved to be effective against the Gα-dependent signaling of GPR30. Phosphorylation of the cAMP-responsive element binding protein (CREB) by PKA was already prominent in the absence of17β-estradiol implying that in addition PKA is activated by other signaling pathways, too. ATF1, a relative to CREB, is phosphorylated almost to the same extent as CREB after stimulation of TNBC cells with 17β-estradiol. This phosphorylation is even more clearly prevented by the pretreatment with 10^-4^ M estriol.

Electrophoretic shift assays (EMSAs) were performed with a labeled oligonucleotide representing the CRE in the promoter of the cyclin D1 promoter at position -14 to +11 from the transcription start site. In TNBC cells treated with 10^-8^ M 17β-estradiol an oligonucleotide band shifted by about 40 kD increased in intensity. In cells pretreated with 10^-4^ M estriol the amount of shifted oligonucleotide was lower than in cells treated with 17β-estradiol alone. Again, this finding provides evidence, that induction of cyclin D1 expression by 17β-estradiol is GPR30 dependent and prevented by estriol treatment. EMSAs showed an additional band at about 150 kD that was detectable in all samples independent of the various treatments of the TNBC cells. The origin of this band is not yet clear. In supershift experiments using an antibody against phosphorylated CREB this band disappeared from the blot because it was shifted to much higher molecular weight. By this supershift experiment this unknown additional band in EMSAs is at least identified to contain CREB. This band probably arises from CREB complexed with other proteins bound to the oligonucleotide of the cyclin D1 promoter.

Using an oligonucleotide with CRE found further upstream at position –517 to –493 we did not observe a binding of CREB to this oligonucleotide at any treatment condition (data not shown).

RT-PCR examinations of mRNA from estriol/estradiol treated cells for cyclin D1 expression confirmed the results of the CREB binding to a cAMP-responsive element of the cyclin D1 promoter, as observed in EMSA tests (Figure 7). All the above mentioned results clearly indicate the involvement of GPR30 in the growth regulation of TNBC by 17β-estradiol.

In addition, we analyzed the GPR30 dependent regulation aromatase expression in TNBC cells. Treatment with 17β-estradiol induced aromatase expression in TNBC cell lines and estriol prevented this induction completely (Figure 5). The induction of aromatase expression by 17β-estradiol is in accordance with a report of Lin et al. [20] who observed a novel signaling paradigm in endometrial cancer cells initiated by estrogenic activation of GPR30. They showed that PI3K and MAPK signal transduction cascades activated by GPR30 converge on nuclear hormone receptor SF-1 that modulates transcription of the aromatase gene [20]. This novel GPR30/SF-1 pathway increases local concentrations of estrogen, and mediates autocrine proliferative effect on cells expressing GPR30. We hypothesize, that in TNBC cells, an autocrine circuit of 17β-estradiol exists that upregulates its own synthesis via stimulation of GPR30. In detail, 17β-estradiol stimulates aromatase expression via GPR30 and the additionally synthesized 17β-estradiol further stimulates GPR30 exponentially leading to growth stimulation of the TNBC cells. Estriol is capable to disrupt this vicious circle by inhibition of GPR30 as we were able to show that induction of aromatase expression by 17β-estradiol is suppressed by treatment with 10^-4^ M estriol. As final consequence of inhibition of GPR30 activity growth stimulation of TNBC cells by 17β-estradiol is clearly prevented by estriol.

High plasma levels of estriol are detected during pregnancy and women who were multiparous have a more than one-third lower risk of breast cancer [28]. The reduced risk for basal-like breast cancer, the major form of TNBC, observed in women with increasing number of breastfed children might be an additional indication for a protective effect of estriol for TNBC [29]. But it should be considered, that serum-levels at the third trimester of pregnancy were estimated to be at 4×10^-8^ M [30] and this is a 1000 times lower concentration than we applied in the proliferation assay of TNBC cell lines.

Due to its limited solubility in culture medium the effects of estriol could not be further increased. The synthetic GPR30 antagonist G15 was even less soluble in culture medium (maximal solubility 10^-5^ M) and therefore less effective in inhibiting EGF-receptor transactivation (data not shown) and less effective in prevention of proliferation (see Additional file 2).

Other GPR30 antagonists are presently under investigation in several laboratories. In 2011, Dennis et al. described another GPR30 antagonist (G36) having a slightly higher affinity to GPR30 than G15 but no information on the aqueous solubility of this compound was given by the authors [14]. Lappano et al. [31] introduced MIBE, an inhibitor, that acts on both GPR30 and estrogen receptor α [31]. Further a tricarbonyl-Re/Tc(I) chelate with GPR30 antagonistic properties has been described [32]. All these compounds are worth testing their efficiency on TNBC.

While measuring the effects of estriol on GPR30 in TNBC cell lines we were aware that besides ERα and GPR30 (GPER) there is a third receptor for estrogens, ERβ, in many different cell types. In addition, it is frequently described, that ERβ is an opponent of ERα, and from our results we cannot certainly reason that the inhibitory action of estriol in TNBC is solely exerted by GPR30. But Western blots of proteins from our tested cell lines revealed a very low expression of ERβ in these cell lines (see Additional file 3: Figure S3).

Up to now, no targeted therapy for TNBC was shown to be successful. Promising candidates, like PARP inhibitors, taking advantage of a disturbed DNA-repair due to frequent BRCA1-mutations present in TNBC [3, 4] and the use of the EGF-R antibody Cetuximab for therapy targeting the overexpression of the EGF-receptor in TNBC were of limited success [2–4].

Our observation, that growth of TNBC is stimulated by 17β-estradiol via GPR30 indicates that reduction of 17β-estradiol in TNBC patients by application of aromatase inhibitors might remain a therapeutic option in triple-negative breast cancer. The efficacy of estriol in inhibiting proliferation of TNBC cells should be further evaluated in vivo.

## Conclusions

In principle we were able to show that GPR30 is involved in growth stimulation of triple-negative breast cancer by 17β-estradiol. Estriol effectively inhibited signal transduction of GPR30 and successfully prevented growth promotion by 17β-estradiol. These results clearly show that a pharmacological inhibition of GPR30 is a promising targeted treatment option for triple-negative breast cancer. The concentrations of estriol needed for sufficient growth inhibition are unfortunately unphysiologically high. There is a need for developing more effective inhibitors for GPR30.

## Electronic supplementary material

Additional file 1: Figure S1: Growth curves of HCC1806 cells in dependence of 17β-estradiol. HCC1806 cells were grown in phenolred-free medium supplemented with 10% charcoal stripped serum for one to seven days (_•_) control or in the presence of 10^-10^ M 17β-estradiol (■) or in the presence of 10^-9^ M 17β-estradiol (▲). Cells were counted under each condition on day 1; day 2; day 4 and day 7 and related to the cell number (100%) seeded to the six-well plates. The minimal growth of HCC1806 cells in the medium without 17β-estradiol is probably due to growth factors like EGF etc present in the charcoal stripped serum. (PPTX 49 KB)

Additional file 2: Figure S2: Inhibition of growth of HCC1806 cells by GPR30 antagonist G15. HCC1806 cells were grown in phenolred-free medium supplemented with 10% charcoal stripped serum in the presence of increasing concentrations of G15 for 7 days and relative cell number was estimated using colorimetric Alamarblue assay. Aqueous solubility of G15 was limited to 4x10^-5^M G15. Whereas low concentrations of G15 (2x10^-6^ M – 2x10^-5^ M) slightly increased cell growth compared to control in the presence of 4x10^-5^ M G15 cell number of HCC1806 was significantly reduced to about 40% of control. Growth inhibition by G15 could not be further increased due to solubility limits. (PPTX 48 KB)

Additional file 3: Figure S3: Expression of three different receptors for 17β-estradiol in breast cancer cell lines. Western blots of 20 μg protein of four breast cancer cell lines were sequentially analyzed with antibodies for ERα, ERβ and GPR30. All three antigens were highly expressed in MCF-7 cells. Expression of ERα was strong in MCF-7, weak in HCC70 and non-detectable in HCC1806 and MDA-MB-453. ERβ was highly expressed in MCF-7 and negligible in HCC1806, HCC70 and MDA-MB-453. GPR30 expression was visible in all four cell lines. (PPTX 1 MB)

## Abbreviations

EGF: Epidermal growth factor
EMSA: Electrophoretic mobility shift assay
ERα: Estrogen receptor α
Erk: Extracellular signal regulated kinase
FCS: Fetal calf serum
GPR30: G-protein coupled receptor
RT-PCR: Reverse transcription polymerase chain reaction
PARP: Poly-(ADP-ribose) polymerase.

## References

[1] Dowsett M, Cuzick J, Ingle J, Coates A, Forbes J, Bliss J, Buyse M, Baum M, Buzdar A, Colleoni M, Coombes C, Snowdon C, Gnant M, Jakesz R, Kaufmann M, Boccardo F, Godwin J, Davies C, Peto R (2010). Meta-analysis of breast cancer outcomes in adjuvant trials of aromatase inhibitors versus tamoxifen. J Clin Oncol.

[2] Carey LA, Dees EC, Sawyer L, Gatti L, Moore DT, Collichio F, Ollila DW, Sartor CI, Graham ML, Perou CM (2007). The triple negative paradox: primary tumor chemosensitivity of breast cancer subtypes. Clin Cancer Res.

[3] Anders CK, Winer EP, Ford JM, Dent R, Silver DP, Sledge GW, Carey LA (2010). Poly(ADP-Ribose) polymerase inhibition: “targeted” therapy for triple-negative breast cancer. Clin Cancer Res.

[4] Telli ML, Ford JM (2010). PARP inhibitors in breast cancer. Clin Adv Hematol Oncol.

[5] Filardo EJ, Quinn JA, Bland KI, Frackelton AR (2000). Estrogen-induced activation of Erk-1 and Erk-2 requires the G protein-coupled receptor homolog, GPR30, and occurs via trans-activation of the epidermal growth factor receptor through release of HB-EGF. Mol Endocrinol.

[6] Revankar CM, Cimino DF, Sklar LA, Arterburn JB, Prossnitz ER (2005). A transmembrane intracellular estrogen receptor mediates rapid cell signaling. Science.

[7] Razandi M, Oh P, Pedram A, Schnitzer J, Levin ER (2002). ERs associate with and regulate the production of caveolin: implications for signaling and cellular actions. Mol Endocrinol.

[8] Steiman J, Peralta EA, Louis S, Kamel O (2013). Biology of the estrogen receptor, GPR30, in triple negative breast cancer. Am J Surg.

[9] Aronica SM, Kraus WL, Katzenellenbogen BS (1994). Estrogen action via the cAMP signaling pathway: stimulation of adenylate cyclase and cAMP-regulated gene transcription. Proc Natl Acad Sci U S A.

[10] Visram H, Greer PA (2006). 17beta-estradiol and tamoxifen stimulate rapid and transient ERK activationin MCF-7 cells via distinct signaling mechanisms. Cancer Biol Ther.

[11] Lazennec G, Thomas JA, Katzenellenbogen BS (2001). Involvement of cyclic AMP response element binding protein (CREB) and estrogen receptor phosphorylation in the synergistic activation of the estrogen receptor by estradiol and protein kinase activators. J Steroid Biochem Mol Biol.

[12] Girgert R, Emons G, Grundker C (2012). Inactivation of GPR30 reduces growth of triple-negative breast cancer cells: possible application in targeted therapy. Breast Cancer Res Treat.

[13] Chen JQ, Russo J (2009). ERalpha-negative and triple negative breast cancer: molecular features and potential therapeutic approaches. Biochim Biophys Acta.

[14] Dennis MK, Field AS, Burai R, Ramesh C, Petrie WK, Bologa CG, Oprea TI, Yamaguchi Y, Hayashi S, Sklar LA, Hathaway HJ, Arterburn JB, Prossnitz ER (2011). Identification of a GPER/GPR30 antagonist with improved estrogen receptor counterselectivity. J Steroid Biochem Mol Biol.

[15] Lappano R, Rosano C, De Marco P, De Francesco EM, Pezzi V, Maggiolini M (2010). Estriol acts as a GPR30 antagonist in estrogen receptor-negative breast cancer cells. Mol Cell Endocrinol.

[16] Girgert R, Bartsch C, Hill SM, Kreienberg R, Hanf V (2003). Tracking the elusive antiestrogenic effect of melatonin: a new methodological approach. Neuro Endocrinol Lett.

[17] Stanley ER, Palmer RE, Sohn U (1977). Development of methods for the quantitative in vitro analysis of androgen-dependent and autonomous Shionogi carcinoma 115 cells. Cell.

[18] Girgert R, Hanf V, Emons G, Grundker C (2009). Membrane-bound melatonin receptor MT1 down-regulates estrogen responsive genes in breast cancer cells. J Pineal Res.

[19] Carmeci C, Thompson DA, Ring HZ, Francke U, Weigel RJ (1997). Identification of a gene (GPR30) with homology to the G-protein-coupled receptor superfamily associated with estrogen receptor expression in breast cancer. Genomics.

[20] Lin BC, Suzawa M, Blind RD, Tobias SC, Bulun SE, Scanlan TS, Ingraham HA (2009). Stimulating the GPR30 estrogen receptor with a novel tamoxifen analogue activates SF-1 and promotes endometrial cell proliferation. Cancer Res.

[21] Andrisani OM (1999). CREB-mediated transcriptional control. Crit Rev Eukaryot Gene Expr.

[22] Wiggin GR, Soloaga A, Foster JM, Murray-Tait V, Cohen P, Arthur JS (2002). MSK1 and MSK2 are required for the mitogen- and stress-induced phosphorylation of CREB and ATF1 in fibroblasts. Mol Cell Biol.

[23] Scaling AL, Prossnitz ER, Hathaway HJ (2014). GPER mediates estrogen-induced signaling and proliferation in human breast epithelial cells and normal and malignant breast. Horm Cancer.

[24] Wang D, Hu L, Zhang G, Zhang L, Chen C (2010). G protein-coupled receptor 30 in tumor development. Endocrine.

[25] Brunello A, Borgato L, Basso U, Lumachi F, Zagonel V (2013). Targeted approaches to triple-negative breast cancer: current practice and future directions. Curr Med Chem.

[26] Lappano R, Pisano A, Maggiolini M (2014). GPER Function in Breast Cancer: an overview. Front Endocrinol (Lausanne).

[27] Smith HO, Arias-Pulido H, Kuo DY, Howard T, Qualls CR, Lee SJ, Verschraegen CF, Hathaway HJ, Joste NE, Prossnitz ER (2009). GPR30 predicts poor survival for ovarian cancer. Gynecol Oncol.

[28] Jacobson HI, Lemanski N, Agarwal A, Narendran A, Turner KE, Bennett JA, Andersen TT (2010). A proposed unified mechanism for the reduction of human breast cancer risk by the hormones of pregnancy. Cancer Prev Res (Phila).

[29] Millikan RC, Newman B, Tse CK, Moorman PG, Conway K, Dressler LG, Smith LV, Labbok MH, Geradts J, Bensen JT, Jackson S, Nyante S, Livasy C, Carey L, Earp HS, Perou CM (2008). Epidemiology of basal-like breast cancer. Breast Cancer Res Treat.

[30] Goodwin TM (1999). A role for estriol in human labor, term and preterm. Am J Obstet Gynecol.

[31] Lappano R, Santolla MF, Pupo M, Sinicropi MS, Caruso A, Rosano C, Maggiolini M (2012). MIBE acts as antagonist ligand of both estrogen receptor alpha and GPER in breast cancer cells. Breast Cancer Res.

[32] Burai R, Ramesh C, Nayak TK, Dennis MK, Bryant BK, Prossnitz ER, Arterburn JB (2012). Synthesis and characterization of tricarbonyl-Re/Tc(I) chelate probes targeting the G protein-coupled estrogen receptor GPER/GPR30. PLoS One.

[CR33] The pre-publication history for this paper can be accessed here: http://www.biomedcentral.com/1471-2407/14/935/prepub

