# Supplementary figures and images for: Inhibition of GPR30 by estriol prevents growth stimulation of triple-negative breast cancer cells by 17β-estradiol
Source: BMC Cancer. 2014 Dec 11;14:935. doi: 10.1186/1471-2407-14-935 (PMC4364648; doi:10.1186/1471-2407-14-935)

## Slide 1
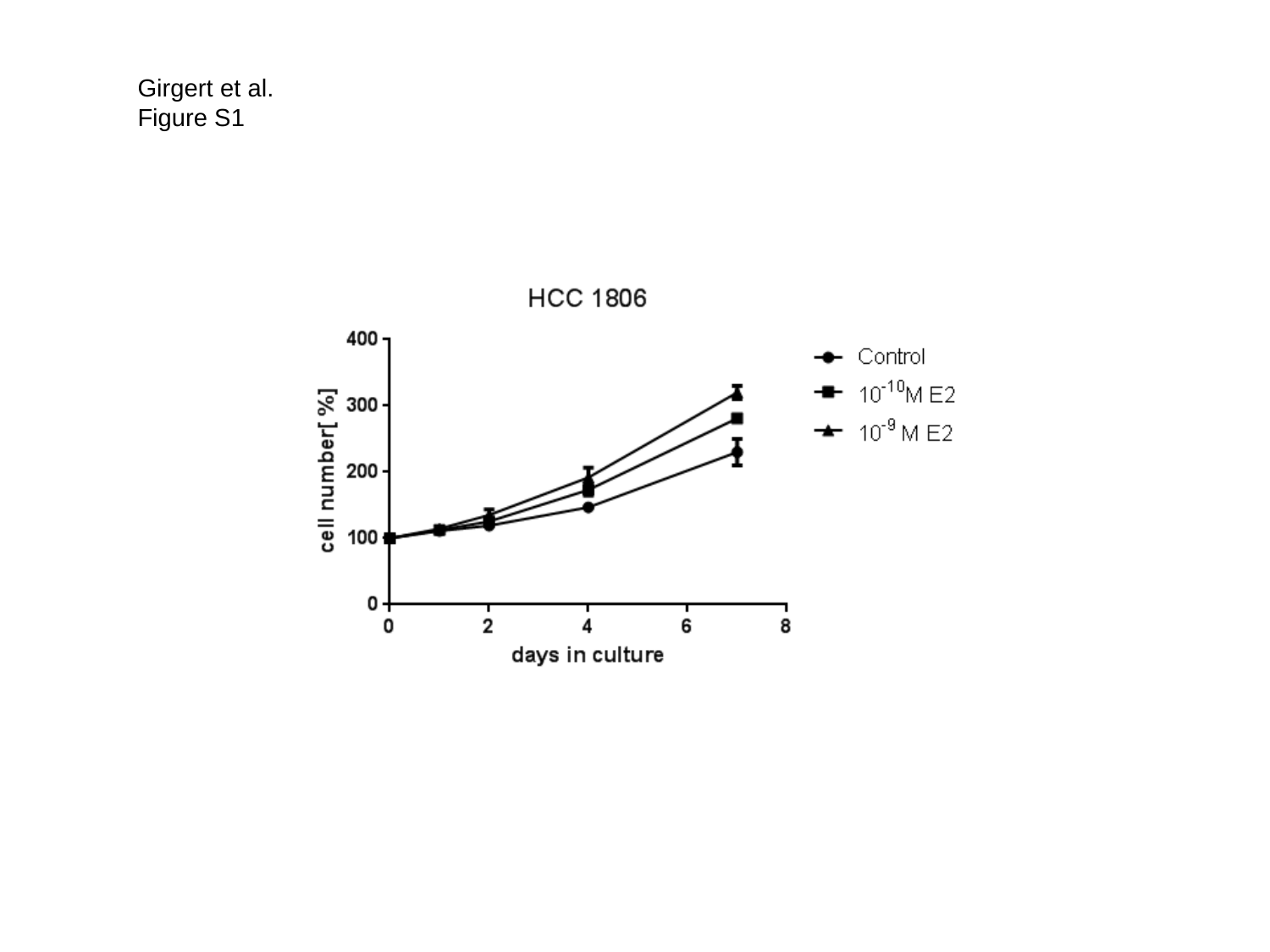

Girgert et al.
Figure S1

Supplement: Supplementary file 1 — Additional file 1: Figure S1: Growth curves of HCC1806 cells in dependence of 17β-estradiol. HCC1806 cells were grown in phenolred-free medium supplemented with 10% charcoal stripped serum for one to seven days (•) control or in the presence of 10-10 M 17β-estradiol (■) or in the presence of 10-9 M 17β-estradiol (▲). Cells were counted under each condition on day 1; day 2; day 4 and day 7 and related to the cell number (100%) seeded to the six-well plates. The minimal growth of HCC1806 cells in the medium without 17β-estradiol is probably due to growth factors like EGF etc present in the charcoal stripped serum. (PPTX 49 KB) [file 12885_2014_5176_MOESM1_ESM.pptx]

## Slide 1
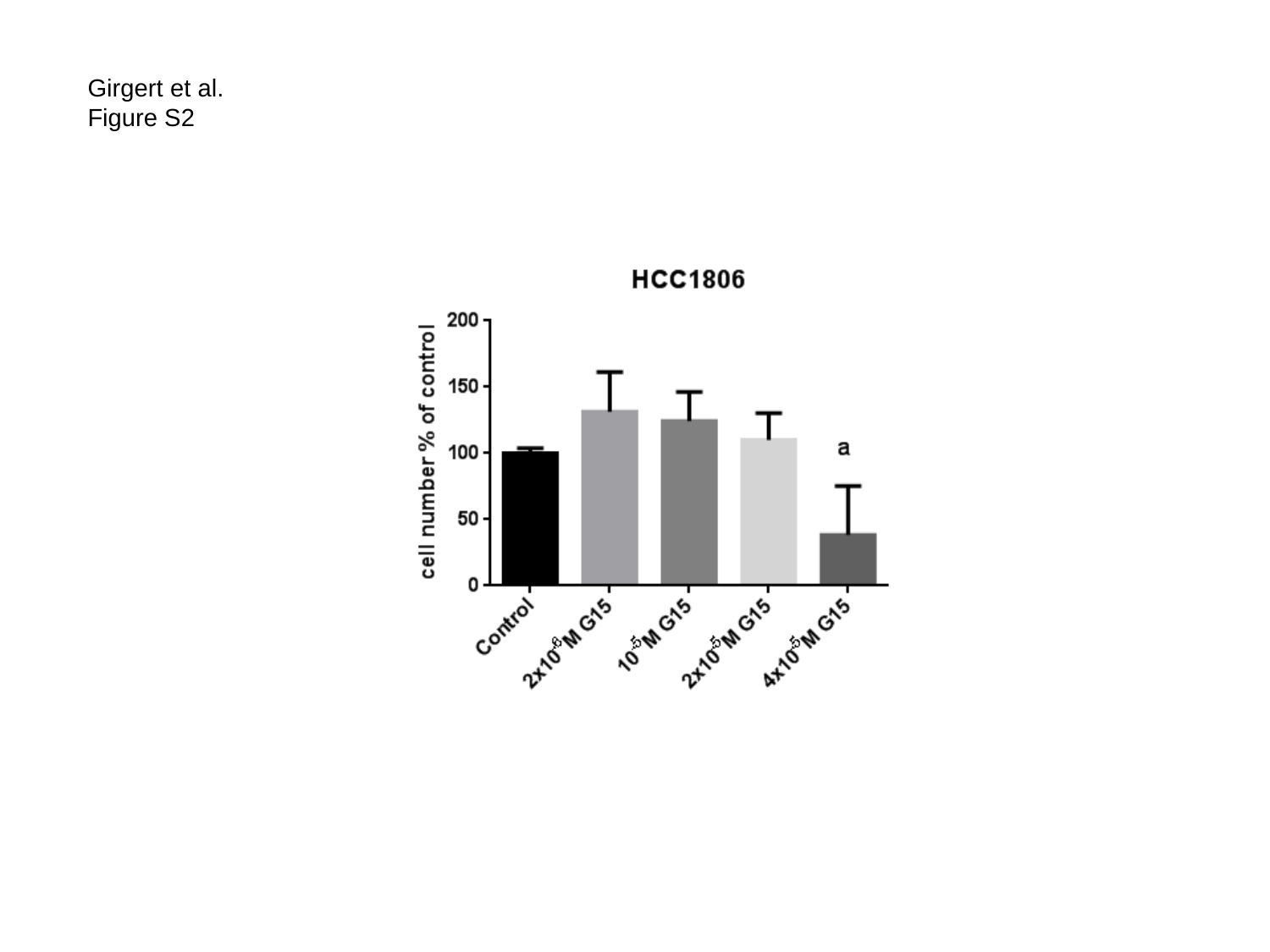

Girgert et al.
Figure S2

Supplement: Supplementary file 2 — Additional file 2: Figure S2: Inhibition of growth of HCC1806 cells by GPR30 antagonist G15. HCC1806 cells were grown in phenolred-free medium supplemented with 10% charcoal stripped serum in the presence of increasing concentrations of G15 for 7 days and relative cell number was estimated using colorimetric Alamarblue assay. Aqueous solubility of G15 was limited to 4x10^-5^M G15. Whereas low concentrations of G15 (2x10^-6^ M – 2x10^-5^ M) slightly increased cell growth compared to control in the presence of 4x10^-5^ M G15 cell number of HCC1806 was significantly reduced to about 40% of control. Growth inhibition by G15 could not be further increased due to solubility limits. (PPTX 48 KB) [file 12885_2014_5176_MOESM2_ESM.pptx]

## Slide 1
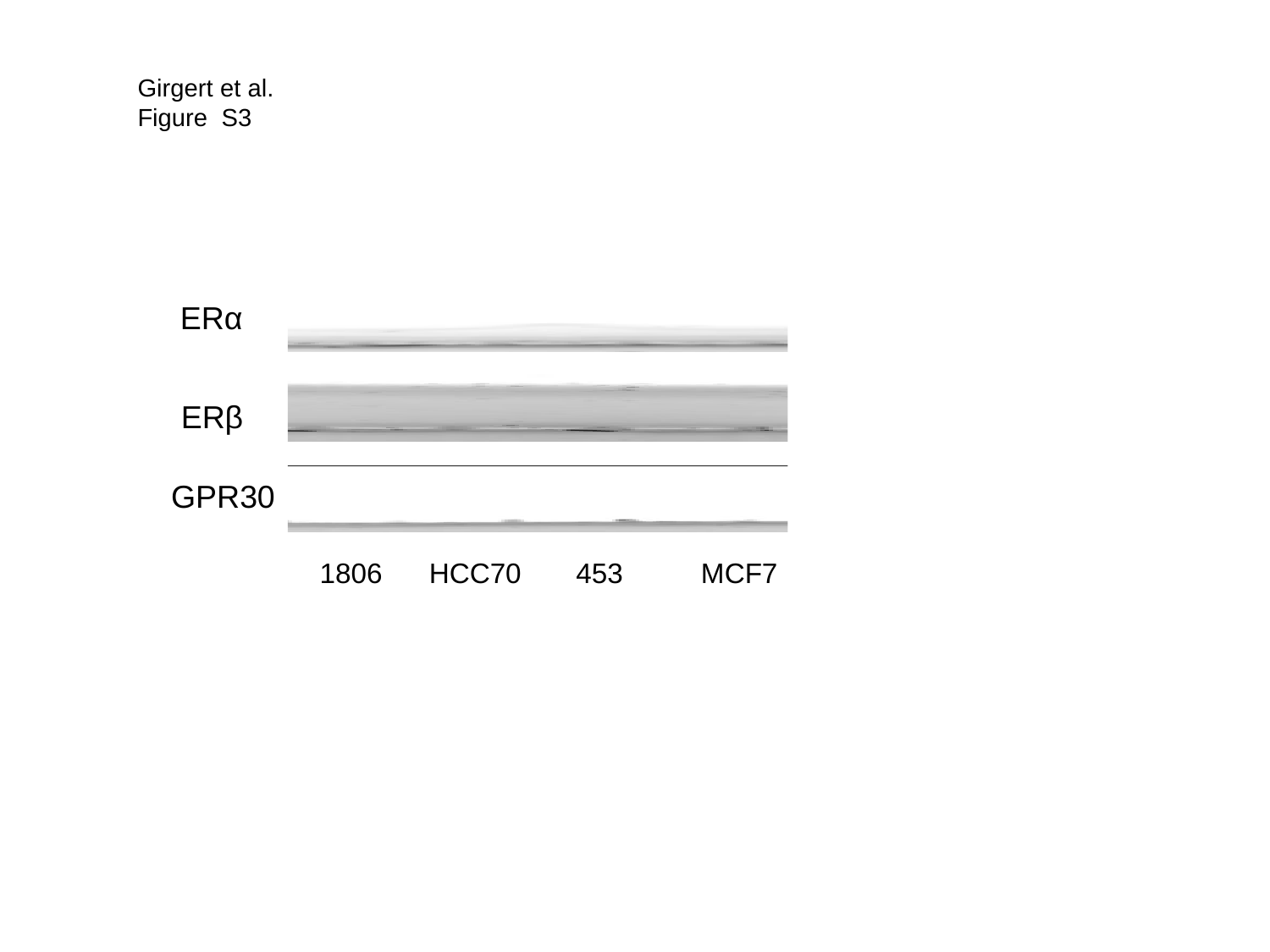

Girgert et al.
Figure S3
ERα
ERβ
GPR30
 1806 HCC70 453 MCF7

Supplement: Supplementary file 3 — Additional file 3: Figure S3: Expression of three different receptors for 17β-estradiol in breast cancer cell lines. Western blots of 20 μg protein of four breast cancer cell lines were sequentially analyzed with antibodies for ERα, ERβ and GPR30. All three antigens were highly expressed in MCF-7 cells. Expression of ERα was strong in MCF-7, weak in HCC70 and non-detectable in HCC1806 and MDA-MB-453. ERβ was highly expressed in MCF-7 and negligible in HCC1806, HCC70 and MDA-MB-453. GPR30 expression was visible in all four cell lines. (PPTX 1 MB) [file 12885_2014_5176_MOESM3_ESM.pptx]
